# Supplementary material for: The effects of age on resting-state BOLD signal variability is explained by cardiovascular and cerebrovascular factors
Source: Psychophysiology. Author manuscript; Available in PMC 2021 Aug 18. (PMC8244027; doi:10.1111/psyp.13714)
Supplement: Supplementary Material [file EMS112773-supplement-Supplementary_Material.pdf]

9. Supplementary Figures

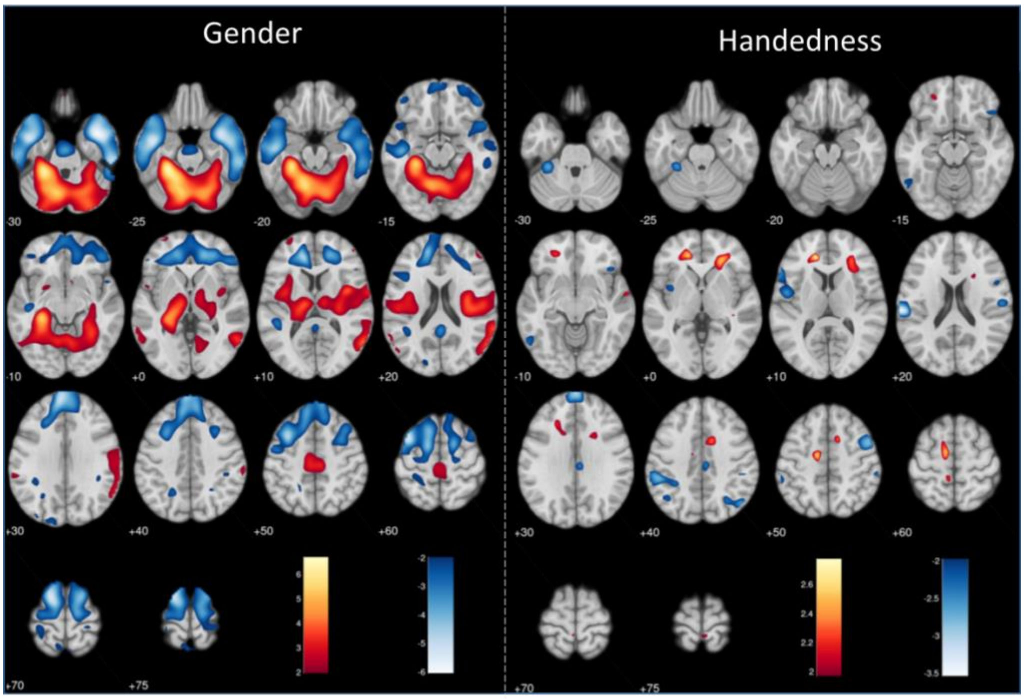

Supplementary Figure 1. Voxel-wise associations between RSFA and covariates of no interests (gender – left panel and handedness – right panel), Model I. Maps are thresholded at uncorrected p-values of 0.05 for more complete description of the spatial represnation.

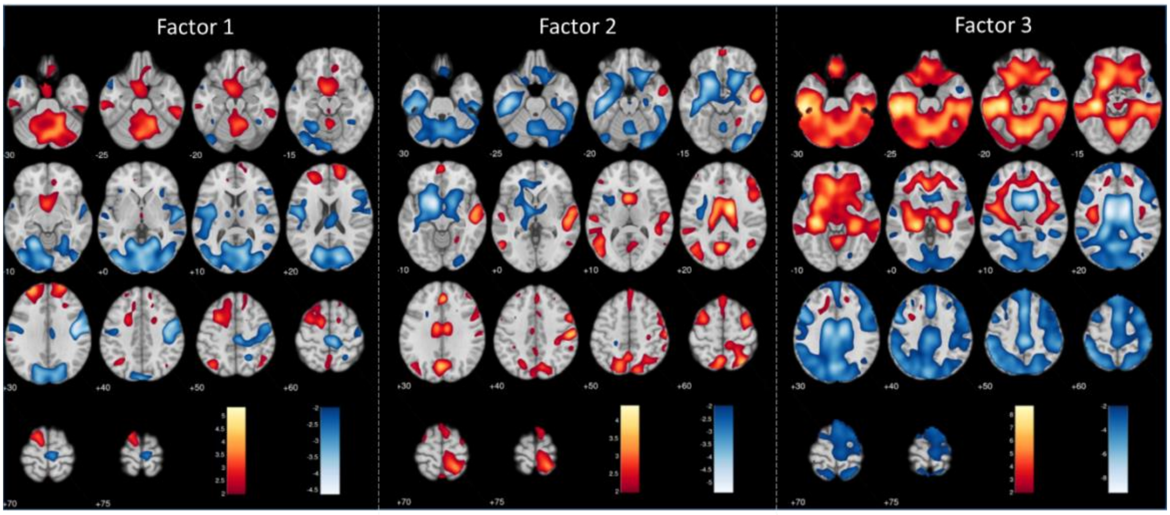

Supplementary Figure 2. Voxel-wise associations between RSFA and three factors of cardiovascular health (Model III). Maps are thresholded at uncorrected p-values of 0.05 for more complete description of the spatial represnation.

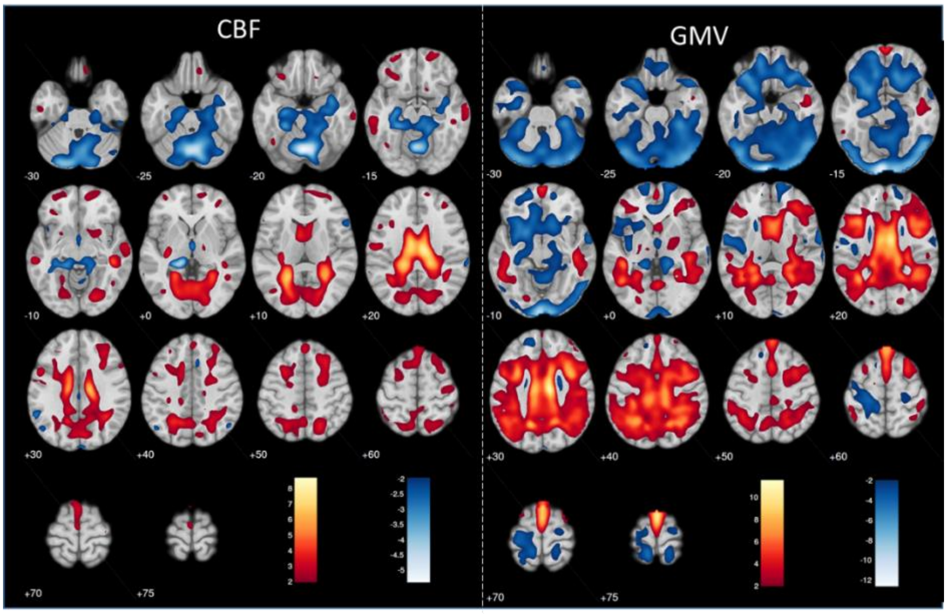

Supplementary Figure 3. Voxel-wise associations between RSFA and CBF (left panel, Model II) and GMV (right panel, Model V). Maps are thresholded at uncorrected p-values of 0.05 for more complete description of the spatial represnation.
