# Supplementary material for: The effects of age on resting‐state BOLD signal variability is explained by cardiovascular and cerebrovascular factors
Source: Psychophysiology. 2020 Nov 18;58(7):e13714. doi: 10.1111/psyp.13714 (PMC8244027; doi:10.1111/psyp.13714)
Supplement: Supplementary file 1 [file PSYP-58-e13714-s001.docx]

**The effects of age on resting-state BOLD signal variability is explained by cardiovascular and cerebrovascular factors**

**Kamen A. Tsvetanov**^**1,2,***^**,** **Richard N.A. Henson**^**3,4**^**,** **P. Simon Jones**^**2**^**,** **Henk-Jan Mutsaerts**^**5**^, **Delia Fuhrmann**^**3**^, **Lorraine K. Tyler**^2^, **Cam-CAN**^**1,2**^ **and** **JAMES B. ROWE**^2,3^

** Corresponding author (*[kat35@cam.ac.uk](mailto:kat35@cam.ac.uk)***, +44 1223 766 556****)*

^*1*^ *Department of Clinical Neurosciences, University of Cambridge, Cambridge, UK* 

 ^*2*^ *Centre for Speech, Language and the Brain, Department of Psychology, University of Cambridge, Cambridge, UK* 

^*3*^ *Medical Research Council Cognition and Brain Sciences Unit, Cambridge, UK* 

^*4*^ *Department of Psychiatry, University of Cambridge, Cambridge, UK*

^*5*^ *Department of Radiology and nuclear medicine, Amsterdam University Medical Center, Amsterdam, The Netherlands* 

# 1. Supplementary Figures

Supplementary Figure 1. Voxel-wise associations between RSFA and covariates of no interests (gender – left panel and handedness – right panel), Model I. Maps are thresholded at uncorrected p-values of 0.05 for more complete description of the spatial represnation.

Supplementary Figure 2. Voxel-wise associations between RSFA and three factors of cardiovascular health (Model III). Maps are thresholded at uncorrected p-values of 0.05 for more complete description of the spatial represnation.

 

Supplementary Figure 3. Voxel-wise associations between RSFA and CBF (left panel, Model II) and GMV (right panel, Model V). Maps are thresholded at uncorrected p-values of 0.05  for more complete description of the spatial represnation.
